# Association of atrial mechanical dispersion with atrial fibrillation recurrence following catheter ablation: results of the ASTRA-AF pilot study

**DOI:** 10.1007/s00392-024-02435-0

**Published:** 2024-05-21

**Authors:** Dorit Knappe, Julia Vogler, Jessica Weimann, Victor Banas, Sevenai Yildirim, Felix Memenga, Juliana Senftinger, Laura Keil, Djemail Ismaili, Moritz Nies, Andreas Rillig, Stephan Willems, Stefan Blankenberg, Paulus Kirchhof, Andreas Metzner, Christoph Sinning

**Affiliations:** 1 Department of Cardiology, University Heart and Vascular Center Hamburg, Martinistraße 52, 20251 Hamburg, Germany; 2 Department of Rhythmology, University Heart Center Lübeck, Lübeck, Germany; 3 Department of Medicine III (Cardiology, Angiology, Intensive Care Medicine), University Hospital Schleswig-Holstein, Kiel, Germany; 4 Department of Cardiology and Internal Intensive Care Medicine, Asklepios Hospital St. Georg, Hamburg, Germany; 5 German Center for Cardiovascular Research (DZHK), Partner Site Hamburg/Kiel/Lübeck, Hamburg, Germany; 6 Institute of Cardiovascular Sciences and SWBH and UHB NHS Trusts, Birmingham, UK

**Keywords:** Atrial fibrillation, Echocardiography, Atrial mechanical dispersion, Recurrence of atrial fibrillation, Catheter ablation

## Abstract

**Aims:**

For patients with symptomatic drug-refractory atrial fibrillation (AF), catheter ablation to achieve rhythm control is an important therapeutic option. The atrial mechanical dispersion measured as standard deviation of the time to peak strain (SD-TPS) is associated with the risk of AF recurrence following catheter ablation.

**Methods:**

The study cohort prospectively enrolled *n* = 132 consecutive patients with paroxysmal (*n* = 88) or persistent AF (*n* = 44) presenting for de novo pulmonary vein isolation (PVI) and followed for 1 year. We related left atrial (LA) volume, LA ejection fraction, SD-TPS, and global longitudinal strain of the left ventricle and clinical variables (sex, age, and type of AF) to AF recurrence.

**Results:**

Kaplan–Meier curves showed higher AF recurrence rate with an increase of SD-TPS with the calculated cut-off of 38.6 ms. Uni- and multivariable Cox regression analysis could show that SD-TPS had the highest relevance regarding AF recurrence with a HR of 1.05 (95% CI, 1.01; 1.09, *p* = 0.01) and HR of 1.05 (95% CI, 1.01; 1.09, *p* = 0.02) per 10 ms increase. In the additional analyses for the model including the clinical variables age, sex, and type of AF with paroxysmal or persisting AF, SD-TPS did only show a trend and after adjusting for covariates, SD-TPS showed a HR of 1.04 (95% CI, 0.99; 1.09, *p* = 0.09) per 10 ms increase.

**Conclusion:**

Atrial mechanical dispersion was associated with recurrent AF.

**Graphical Abstract:**

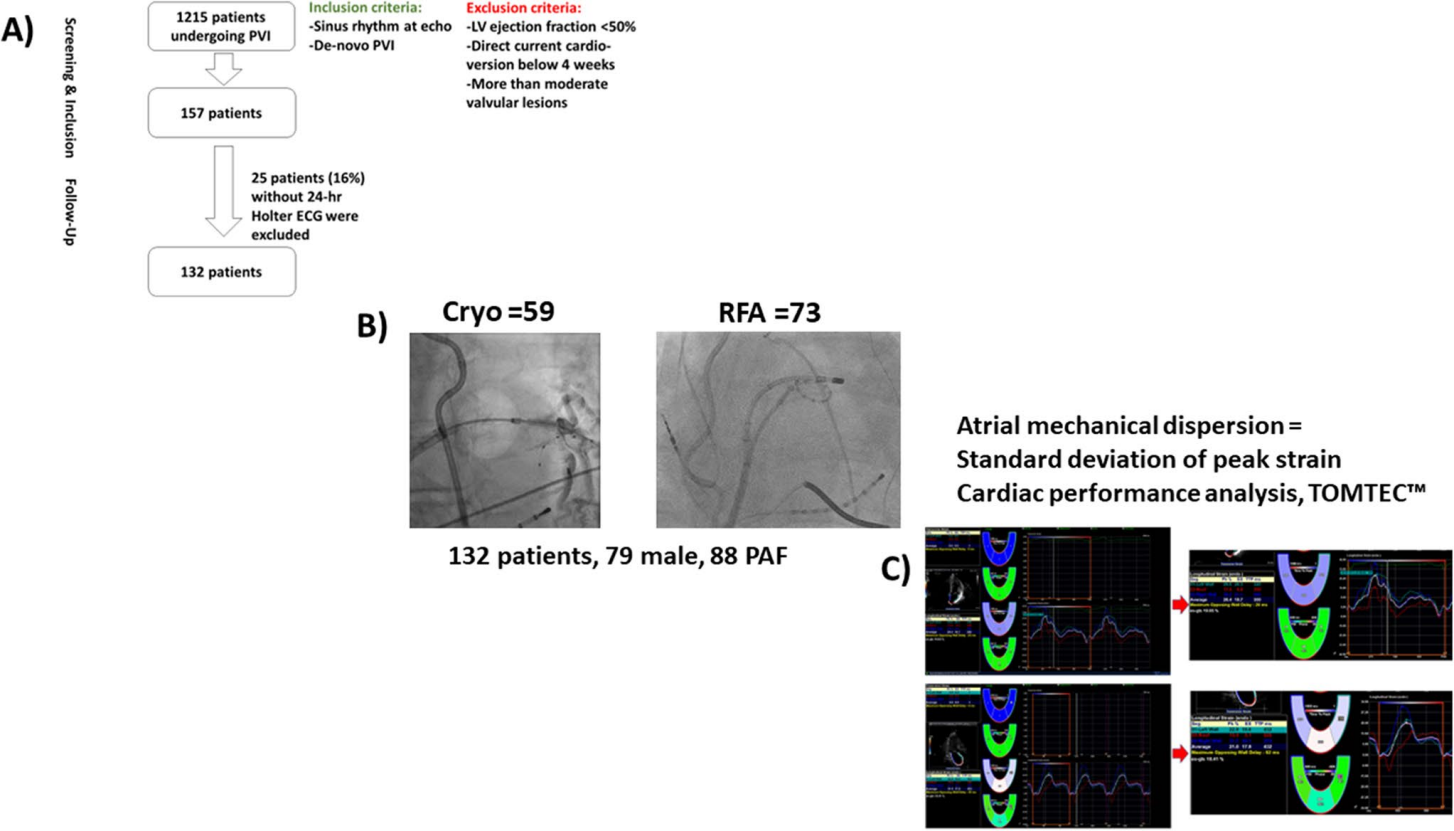

## Introduction

Atrial fibrillation (AF) is the most prevalent cardiac rhythm disorder among the elderly, with an observed increase in mortality rates for men and women aged over 50 and 60 years, respectively, following an AF diagnosis [1, 2]. Recent studies have shown that strategies focusing on rhythm control, particularly early intervention, result in fewer cardiovascular events in AF patients compared to the standard rate control care [3]. In this regard, catheter ablation has proven more effective in maintaining sinus rhythm after 1 year compared to pharmacological treatments, with AF recurrence rates ranging from 25 to 42% [4, 5]. However, it is important to note that while repeat procedures can improve success rates, they also come with higher costs and an increased likelihood of adverse events [1]. Therefore, careful evaluation using additional diagnostic tests is essential when considering catheter ablation for patients. The recent guidelines recommend using transthoracic echocardiography to detect structural cardiac changes associated with AF [1]. As AF is a progressive disease which facilitates its own persistence due to atrial remodeling, left atrial (LA) volume enlargement is a useful parameter to predict AF recurrence [6, 7]. When considering LA remodeling, strain echocardiography can evaluate the local myocardial function, and a decrease in LA strain is linked to the occurrence of AF in both the general population and patients with ischemic stroke [8, 9]. Myocardial strain is as well suitable to evaluate the timing of myocardial contraction, and there is a reported association between left ventricular (LV) mechanical dispersion and the occurrence of arrhythmias [10]. However, it has also been observed that disturbances in the timing of LA contraction indicate the presence of fibrosis and electrophysiological disorders, which may suggest a high likelihood of AF recurrence, such as intra-atrial dyssynchrony during sinus rhythm as a specific disorder [11]. Recently, a study described the additional value of measuring LA mechanical dispersion as standard deviation of the time to peak strain (SD-TPS) to evaluate the risk of AF recurrence in subjects at risk of developing heart failure [12].

The primary objective of the present study is (1) to examine the association between SD-TPS, a marker of LA mechanical dispersion, and AF recurrence. Additionally, the study aims (2) to assess the influence of SD-TPS in conjunction with other recently proposed imaging variables and clinical factors such as sex, age, and type of AF. These variables are commonly evaluated in various models of AF recurrence after catheter ablation [13].

## Methods

The study cohort consisted of 132 patients presenting with AF for the first catheter ablation procedure to the University Heart & Vascular Center Hamburg, Hamburg, Germany, from December 2017 to January 2019. The patients were included into the single-center, observational ASTRA-AF (Left Atrial STRAin in Patients undergoing Atrial Fibrillation Ablation and Recurrence of Arrhythmia) pilot study (reference number at the ethical commission of the Hamburg Medical Association: PV5835). All patients received transthoracic and transesophageal echocardiography prior to catheter ablation. Patients with atrial fibrillation at presentation, poor 2D imaging quality, or impaired left ventricular ejection fraction (< 50%) were excluded. In addition, patients with moderate or severe valvular heart disease were excluded as well. Patients were treated with either radiofrequency ablation (RFA) or cryoballoon (Cryo) ablation at the discretion of the treating physician. Thus, out of 88 patients with paroxysmal AF (PFA), 40 did receive a RFA and 48 a Cryo ablation and in the 44 patients with persistent AF (PersAF), 33 did receive a RFA and 11 a Cryo ablation. In general, following the publication of previous results of CHASE-AF (Catheter Ablation of Persistent Atrial Fibrillation), the approach favored at the center was to do PVI only [14]. Two patients in the cohort with AF recurrence (one with PAF, one with PersAF) within 1 year were treated with additional linear lesions or CFAE ablation.

**Fig. 1:**
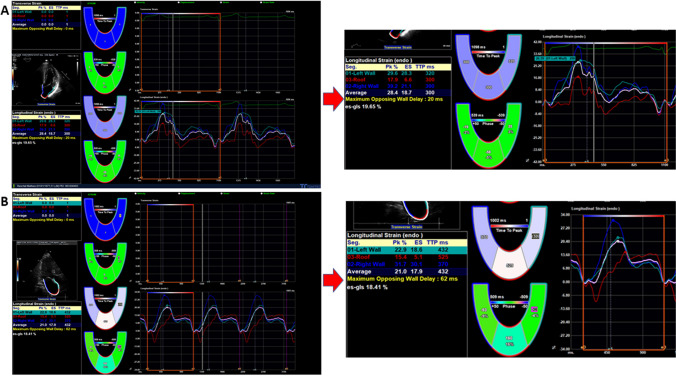
**1A** shows a patient with a low SD-TPS (9.4 ms) regarding the standard deviation of the three segments of the left and right atrial wall and the roof of the atrium. **1B** shows a patient with different time to peak and increased SD-TPS (63.7 ms)

After manual tracing of the LA endocardial border, the software Cardiac Performance Analysis (IMAGE-COM, TOMTEC-ARENA, TomTec Imaging System GmbH, Unterschleissheim, Germany) automatically tracked the myocardium throughout the cardiac cycle. The strain curves of the global and regional LA wall were automatically generated by the software, and the reference point for image analysis was taken at the onset of the QRS complex (R-R gating). LA mechanical dispersion was defined as the standard deviation of time to peak positive strain (SD-TPS) from the three LA segments used by the software. The measurement technique was described previously (Fig. 1) [12]. The LA ejection fraction is determined by the formula: Total emptying fraction (LAV_max_ − LAV_min_) / LAV_max_ [15]. The additional echocardiography included an assessment of diastolic function, measurement of ventricular diameters and volumes, and a three-dimensional assessment of the left ventricle. All measurements, including strain measurements, were performed according to the current recommendations [16] [17]. The baseline data included the type of antiarrhythmic medication and anticoagulation. The CHA_2_DS_2_-VASc score was calculated according to current guideline suggestions [1]. The blanking time was the first 3 months following ablation. After 12 months, all patients were invited to undergo additional transthoracic echocardiography and a 24-h Holter ECG to assess recurrence of AF, current antiarrhythmic medication, and anticoagulation. Further, presence of stroke or need for pacemaker therapy was assessed. Patients who could not attend the follow-up visit were interviewed with a questionnaire and asked to send a current 24-h Holter ECG.

### Statistics

Continuous variables are shown as medians (25th percentile–75th percentile) and compared using the Mann–Whitney *U* test. Binary variables are shown as counts (frequencies) and compared using the *χ*^2^ test. As this is an explorative study, no adjustments for multiple testing have been done. *p*-values are given for descriptive reasons only [18]. The median follow-up time was estimated by the reverse Kaplan–Meier estimator, and the event rate was estimated by the Kaplan–Meier method. The primary endpoint is censored at 1 year of follow-up.

Optimal cut-offs are determined using Cox regression and the function *cutp* from package *survMisc* to make the choice [19].

Determined groups are analyzed within survival analysis in Kaplan–Meier curves. Groups are compared using the log-rank test.

Univariable Cox regression was performed to identify the association of patient characteristics with the recurrence of AF. All variables with a *p*-value < 0.25 in univariable regression were chosen for a multivariable model. Hazard ratios (HR) and corresponding 95% confidence intervals (CI) are given. Forest plots display the respective results. The vertical line at an HR of one is the line of no effect.

A *p*-value of < 0.05 was considered statistically significant in regression analysis. All analyses rely on complete cases and were performed with R statistical software version 4.0.3 (R Foundation for Statistical Computing, Vienna, Austria).

## Results

### Baseline characteristics

The study cohort consisted of 132 patients (79 males, 59.8%), median age was 65.5 years (interquartile range IQR, 55–73), and median CHA_2_DS_2_-VASc score was 2 (IQR, 1–3). The patient cohort consisted of 102 (77.3%) patients without and 30 (22.7%) patients with AF recurrence during follow-up (Table 1). Thirteen patients (9.8%) had a history of ischemic stroke and 13 (9.8%) a history of coronary artery disease.

**Table 1:** Baseline characteristics of the cohort with and without AF recurrence

Variable	Without AF recurrence (*n* = 102)	With AF recurrence (*n* = 30)	*p*-value
Men no. (%)	62 (60.8)	17 (56.7)	0.85
Age (years)	62.0 (54.9, 69.1)	75.0 (57.9, 78.0)	< 0.001
BMI kg/m^2^	26.3 (24.6, 29.5)	26.2 (23.5, 28.0)	0.26
BSA m^2^	2.0 (1.9, 2.2)	2.0 (1.8, 2.1)	0.11
CHA_2_DS_2_-VASc	2.0 (1.0, 3.0)	3.0 (1.9, 4.1)	< 0.001
Arterial hypertension no. (%)	54 (52.9)	24 (80.0)	0.015
Smoking no. (%)	26 (25.5)	7 (23.3)	1.00
Diabetes no. (%)	5 (4.9)	2 (6.7)	1.00
Dyslipidemia no. (%)	11 (10.9)	7 (23.3)	0.15
Stroke no. (%)	9 (8.8)	4 (13.3)	0.70
Coronary artery disease no. (%)	11 (10.8)	4 (13.3)	0.95
Cardioversion no. (%)	40 (39.2)	17 (56.7)	0.14
Antiarrhythmic medication no. (%)	36 (50.0)	9 (42.9)	0.74
Oral anticoagulation no. (%)	77 (75.5)	26 (86.7)	0.29
Radiofrequency ablation no. (%)	58 (56.9)	15 (50.0)	0.65
Cryoballoon ablation no. (%)	44 (43.1)	15 (50.0)	0.65
Persistent AF no. (%)	34 (33.3)	10 (33.3)	1.00

Regarding cardiovascular risk factors, arterial hypertension was present more often in the cohort with AF recurrence with 24 (80.0%) to 54 (52.9%) patients (*p* = 0.02). Treatment of the patients regarding cardioversion or antiarrhythmic medication was not different in patients with and without recurrence of AF. Oral anticoagulation was prescribed in 77 (75.5%) patients without and 26 (86.7%) patients with AF recurrence (*p* = 0.29). The energy source used for treatment of the patients in the cohort without and with AF recurrence was not different in this cohort (*p* = 0.65 for radiofrequency ablation and cryoballoon ablation).

The baseline characteristics for the cohorts with paroxysmal AF (PAF) and persistent AF (PersAF) are shown in Supplemental Table 1.

### Follow-up and recurrence of AF in the overall cohort

The maximum follow-up time was 3.1 years, and the median follow-up time was 801 (95% CI, 776; 835) days. At the censored follow-up time of 12 months, the AF recurrence rate was 22.8%.

### Imaging variables in the cohort with and without AF recurrence

Regarding the imaging variables in relation to AF recurrence (Table 2), median LAVI was 29.4 mL/m^2^ (IQR, 22.8–35.8) in patients without recurrence and 30.7 mL/m^2^ (IQR, 22.7–35.2) in patients with AF recurrence (*p* = 0.74). LA ejection fraction was 41.8% (IQR, 33.9–50.5%) and 34.0% (IQR, 29.1–43.7) (*p* = 0.03) in patients without and with AF recurrence, respectively. For LV global longitudinal strain, the measurements were − 19.7% (IQR, − 22.9 to − 18.0) and − 19.3% (IQR, − 20.9 to − 17.6) for patients without and with AF recurrence (*p* = 0.15). The measured SD-TPS was significantly different in patients without and with AF recurrence: median 25.3 ms (IQR, 12.7–46.5) vs. 61.1 ms (IQR, 42.6–84.1) (*p* < 0.001).

**Table 2:** Imaging variables of the cohort with and without AF recurrence

Variable	Without AF recurrence (*n* = 102)	With AF recurrence (*n* = 30)	*p*-value
LA volume indexed to BSA (mL/m^2^)	29.4 (22.8, 35.8)	30.7 (22.7, 35.2)	0.74
LV ejection fraction 3d (%)	56.0 (53.3, 60.0)	57.3 (52.0, 62.1)	0.57
LV ejection fraction 2d (Simpson) (%)	59.2 (54.0, 64.3)	59.0 (52.2, 64.1)	0.82
LA ejection fraction 2d (Simpson) (%)	41.8 (33.9, 50.5)	34.0 (29.1, 43.7)	0.030
E/A	1.2 (1.0, 1.5)	1.2 (1.0, 1.8)	0.80
*E*/*e*ʹ (average of septal and lateral tissue Doppler)	8.3 (6.8, 10.1)	9.0 (7.4, 11.1)	0.24
LV global longitudinal strain (%)	− 19.7 (− 22.9, − 18.0)	− 19.3 (− 20.9, − 17.6)	0.15
LV strain 3d (%)	− 18.0 (− 20.3, − 14.5)	− 19.1 (− 23.0, − 15.7)	0.12
SD-TPS (ms)	25.3 (12.7, 46.5)	61.1 (42.6, 84.1)	< 0.001
TTP left wall	400.0 (357.0, 451.0)	385.5 (357.5, 432.6)	0.54
TTP roof	409.0 (341.0, 465.7)	394.5 (320.9, 481.6)	0.68
TTP right wall	400.0 (340.0, 451.0)	384.0 (339.0, 467.1)	0.73
Maximum opposing wall delay	33.0 (19.7, 79.3)	43.0 (31.9, 100.7)	0.072

### Imaging variables in the PAF and PersAF cohort

Imaging variables are presented for the PAF and PersAF cohort in Supplemental Table 2.

The median left ventricular (LV) ejection fraction was 59.0% (IQR, 53.7–64.2). Regarding LA volume, the median LA volume indexed to BSA (LAVI) was 28.9 mL/m^2^ (IQR, 21.2–35.0) in patients with PAF and 30.5 mL/m^2^ (IQR, 25.9–44.2) (*p* = 0.03) in PersAF. Regarding imaging parameters of diastolic dysfunction, median E/A was 1.2 (IQR, 1.0–1.6) without a relevant difference for PAF and PersAF (*p* = 0.09) and the same was shown for median *E*/*e*ʹ with 8.4 (IQR, 6.8–10.5) (*p* = 0.3). The median measurement of LV global longitudinal strain was − 19.7% (IQR, − 22.1 to − 17.9) for PAF and − 19.4% (IQR, − 21.6 to − 17.8%) for PersAF (*p* = 0.2), and median LA ejection fraction was 40.9% (IQR, 32.9–50.1) in the PAF cohort and 40.3% (IQR, 25.7–48.7) (*p* = 0.3) in the PersAF cohort, respectively. Regarding SD-TPS, median measurements were 33.5 ms (IQR, 14.4–49.9) in PAF and 38.7 ms (IQR, 20.6–68.0) in PersAF patients (*p* = 0.09).

### Optimal cut-off selection and results regarding the association of imaging variables with AF recurrence

**Fig. 2:**
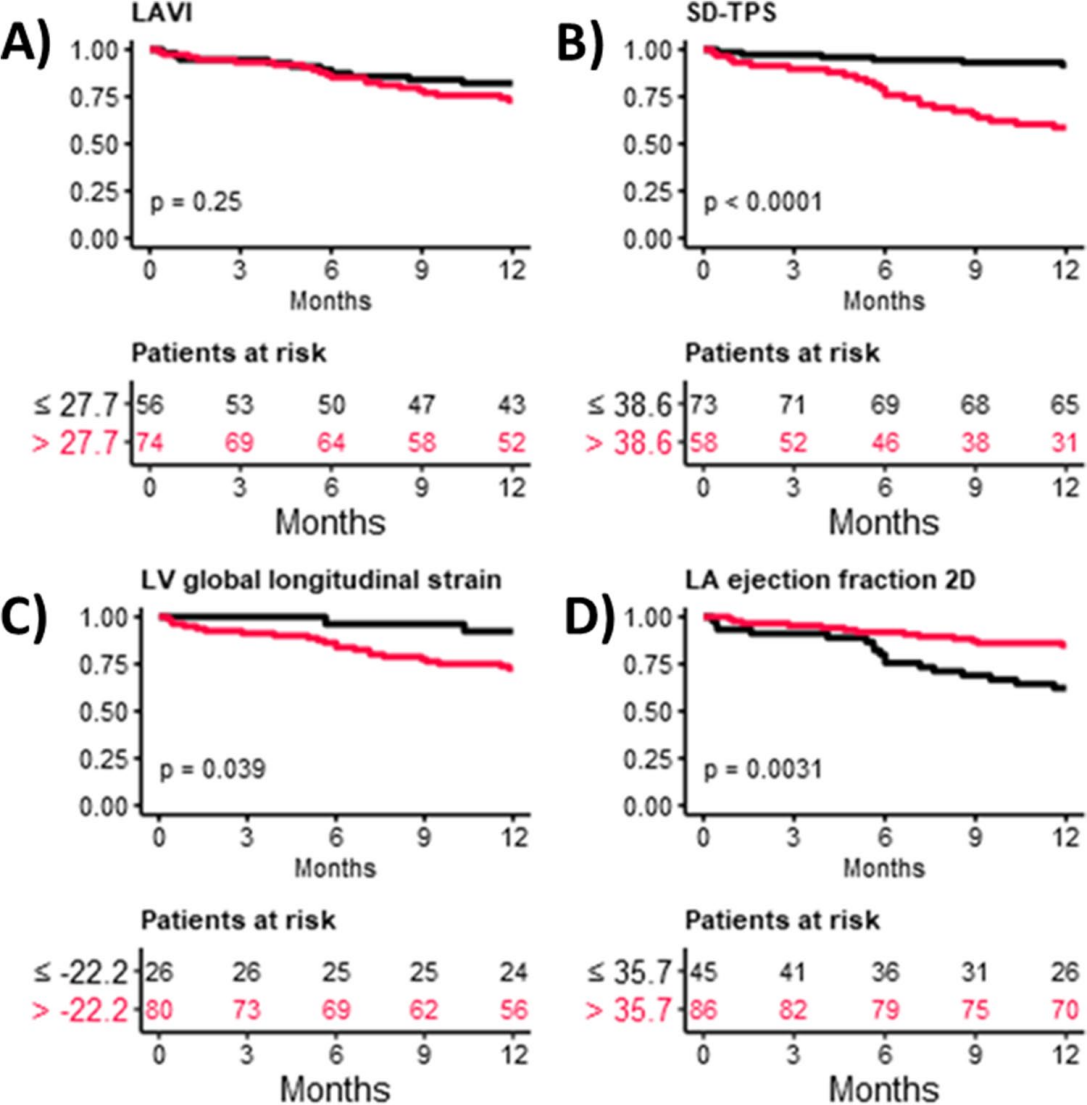
Kaplan–Meier curves regarding the association of imaging variables with the risk of AF recurrence for **A** LAVI, **B** SD-TPS, **C** LV global longitudinal strain, and **D** LAEF

Regarding the imaging variables, optimal cut-offs for the classification of AF recurrence were calculated. The calculated cut-offs for LAVI were 27.7 mL/m^2^, for SD-TPS 38.6 ms, for LV global longitudinal strain − 22.2%, and for LA ejection fraction 35.7%. The cut-off values for SD-TPS (*p* < 0.001), LV global longitudinal strain (*p* = 0.04), and LA ejection fraction (*p* = 0.003) show a relevant association with AF recurrence; however, no relevant association of LAVI with AF recurrence could be established (Fig. 2).

### Uni- and multivariable Cox regression analysis regarding the association with the risk of AF recurrence

**Fig. 3:**
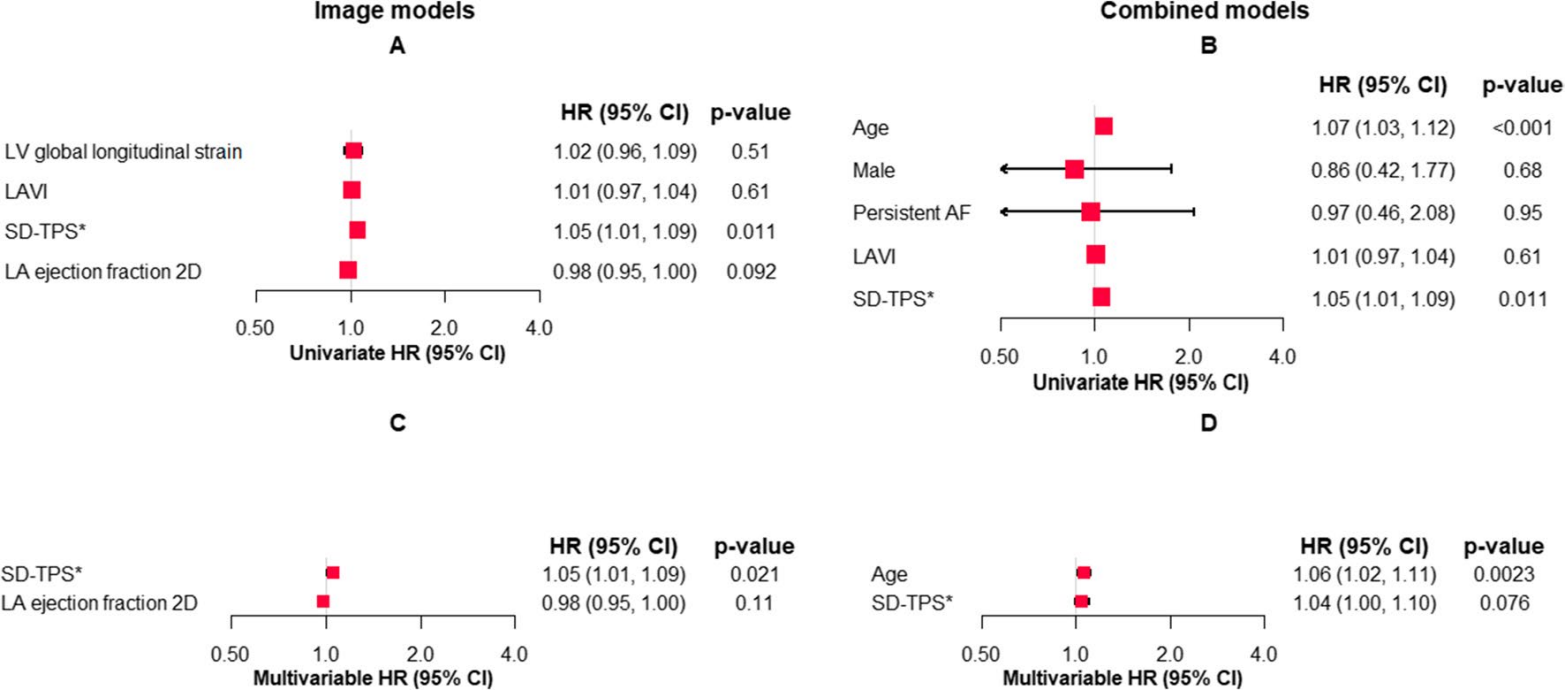
Forest plots showing the results of univariate and multivariable Cox regression analysis regarding the imaging and clinical variables. **A** Univariate analysis for the imaging variables, **B** univariate analysis for the clinical variables, **C** multivariable analysis for the imaging variables, and **D** multivariable analysis including all variables of the clinical model

In univariable Cox regression analysis of the association between imaging variables and AF recurrence, SD-TPS could be identified as a predictor of AF recurrence (hazard ratio [HR] of 1.05, 95% confidence interval [CI] 1.01; 1.09, *p* = 0.01) (Fig. 3). The other imaging variables were not associated with the risk of AF recurrence, and of the clinical variables, only “age” was also predictive of a higher AF recurrence risk. In the multivariable analysis model including the imaging variables, the association with the risk of AF recurrence resulted in a HR of 1.05 (95% CI 1.01; 1.09, *p* = 0.011) for SD-TPS, while the other included variables did not show an association with the risk of the event. In the multivariable analysis including all the clinical variables, “age” was again the only variable predictive of AF recurrence (HR 1.06; 95% CI 1.02, 1.11; *p* = 0.002), while SD-TPS only showed a trend towards higher AF recurrence risk (HR 1.04; 95% CI 1.00, 1.10; *p* = 0.07) (Fig. 3).

## Discussion

In a prospective study utilizing advanced echocardiographic assessments, we have demonstrated the following findings: (1) Atrial mechanical dispersion, as indicated by SD-TPS, is associated with an elevated risk of AF recurrence. (2) On the other hand, left atrial volume index (LAVI) was not associated with the risk of AF recurrence. (3) By establishing a cut-off value of 38.6 ms for SD-TPS, it may be possible to identify patients who are at a higher risk of experiencing AF recurrence. (4) Following catheter ablation using either radiofrequency or cryoballoon techniques, the rate of AF recurrence after 1 year of follow-up was relatively low at 23%. (5) Through multivariable analysis, we determined that age is the most significant clinical variable in terms of the risk of AF recurrence.

### Left atrial structure and risk of AF recurrence

The concept of LV mechanical dispersion assumes that tissue alterations and as a consequence different conduction times can be measured with the timing of the peak strain values, and that increases in the standard deviation of LV mechanical dispersion correlate with the presence of arrhythmias [10]. Further underlining the additional value of measuring SD-TPS in the LA is the previous finding that intra-atrial dyssynchrony during sinus rhythm is associated with an increased risk of AF recurrence following catheter ablation [11]. In our study, in patients both without and with recurrence of AF, mean LAVI was not above the threshold of 34 mL/m^2^, which is often related to diastolic dysfunction, while further increased LA volumes of the LA have long been known to facilitate new episodes of AF and recurrence following a therapeutic intervention [6, 7, 15]. Despite the finding of normal mean LAVIs in the study population, SD-TPS values were higher in patients with AF recurrence in the first year following catheter ablation, suggesting changes of the atrial tissue resulting in a conduction delay which might be a characteristic of developing or present atrial cardiomyopathy [20]. A previous study demonstrated as well that SD-TPS is a marker for recurrence of AF following catheter ablation [21]. However, this study only included patients with PAF, and catheter ablation was performed with radiofrequency ablation in all patients [21]; thus, the presented study more accurately represents a real-world population presenting for catheter ablation [1]. The calculated cut-off value for SD-TPS of 38.6 ms is close to the previously published mean SD-TPS of 38 ms in patients with AF recurrence [21]; however, the cut-off originating from our cohort is data-driven and thus cannot be recommended for general use. The results show that even in a cohort including PAF and PersAF with normal LAVI SD-TPS can be applied to detect changes of the atrial tissue facilitating AF recurrence in terms of prolongation of the SD-TPS [12, 21].

### Imaging variables and recurrence of AF following PVI

In the study, additional imaging variables were assessed as well, including LV global longitudinal strain (LV-GLS), which was reported to be associated with the presence of AF in patients with cryptogenic stroke [22]. However, despite different mean values in the group of patients with and without AF recurrence, LV-GLS was not associated with the risk of AF recurrence in the multivariable analysis. Regarding the LV-GLS with the suggested cut-off from our analysis of − 22.2%, this value is more negative than the suggested − 20% for a healthy individual, which might not reflect the comorbidities of most of the patients [16, 22]. This might add to the finding that functions of the LV and LA have a close relation with influence of the hemodynamic status of the LV on the LA, thus facilitating AF recurrence. The suggested LA ejection fraction is a parameter which is calculated according to the formula as equivalent to the LV ejection fraction [16] and is termed total emptying fraction [23], reflecting the global function of the LA with a reservoir, conduit, and booster pump, and thus might be an indicator of a poor outcome in AF patients. Our study calculated LA ejection fraction with a cut-off of 35.7% lower than the suggested threshold in the literature; however, this might be due to a diversity of the study cohorts; the values were derived from [15]. Left atrial ejection fraction showed a different distribution between patients with and without AF recurrence and lower values in patients with AF recurrence, indicating a reduced total emptying fraction in this group of patients which might as well indicate a decreased function of the LA. However, in multivariable analysis, only SD-TPS was identified as the imaging variable associated with AF recurrence.

### Clinical variables and recurrence of AF following catheter ablation

The variables with the highest predictive value regarding the development of AF are age and sex [1, 2], which have been well studied in different projects researching recurrence of AF following catheter ablation [13]. It is furthermore well established that the type of AF (paroxysmal vs. persistent), reflecting alterations of the atrial myocardial tissue, affects AF recurrence [13, 20].

### Appraisal of the combined approach of combining imaging and clinical variables

The major benefit of combining imaging variables and clinical variables to predict risk of AF recurrence following treatment with catheter ablation is the high availability of data, as echocardiographic parameters are guideline-recommended with class I according to the current guidelines in AF patients [1]. The clinical variables age, sex, and type of AF are routinely collected and available in all patients. Additional suggestions like the inclusion of biomarkers in these models might as well refine the risk assessment of AF recurrence but demand additional resources of the health care system and might thus not be as broadly available [13]. In the multivariable analysis including the clinical variables and SD-TPS, only age remained a predictor of AF recurrence. However, as age is a non-influenceable factor in the treatment of AF, SD-TPS might be a novel diagnostic tool to facilitate treatment decision in AF patients.

Although in current guidelines and in literature LAVI is the preferred imaging variable used for risk stratification regarding development of AF and for treatment decisions, our study demonstrates that consideration of additional imaging variables like atrial mechanical dispersion parameters might be worthwhile in an integrated approach including clinical variables to assess the risk of AF recurrence following catheter ablation.

Defining variables that are reliable for identifying patients at high risk of developing AF following catheter ablation might have implications for increasing screening for AF recurrence [24].

### Strengths and limitations

The major strength of the presented study is that it reflects a real-world cohort of AF patients presenting for the first PVI using state-of-the-art diagnostic and treatment methods with both radiofrequency and cryoballoon ablation. This sets it apart from previous studies as it encompasses patients with both paroxysmal and persisting AF who were candidates for catheter ablation. Additionally, the echocardiographic assessments were conducted by experienced physicians in a large tertiary center. However, it is important to note that the study’s limitations include its single-center nature and small sample size, which necessitates validation of the data-driven results in larger study cohorts. Furthermore, the assessment of SD-TPS requires expertise in interpretation, which may limit its use in general settings.

## Conclusion

The study provides valuable real-world insights into the relationship between novel imaging parameters measuring atrial mechanical dispersion, along with clinical variables, and the risk of AF recurrence after catheter ablation. However, as the data is hypothesis generating, additional validation and iteration in larger cohorts is needed.
